# Yeast Killer Toxin K28: Biology and Unique Strategy of Host Cell Intoxication and Killing

**DOI:** 10.3390/toxins9100333

**Published:** 2017-10-20

**Authors:** Björn Becker, Manfred J. Schmitt

**Affiliations:** Molecular and Cell Biology, Department of Biosciences and Center of Human and Molecular Biology (ZHMB), Saarland University, D-66123 Saarbrücken, Germany; bjoern_becker2@gmx.de

**Keywords:** K28, killer toxin, *S. cerevisiae*, A/B toxin, cell wall receptor, H/KDEL receptor, retrograde protein transport, retrotranslocation, cell cycle arrest, toxin immunity

## Abstract

The initial discovery of killer toxin-secreting brewery strains of *Saccharomyces cerevisiae* (*S. cerevisiae*) in the mid-sixties of the last century marked the beginning of intensive research in the yeast virology field. So far, four different *S. cerevisiae* killer toxins (K28, K1, K2, and Klus), encoded by cytoplasmic inherited double-stranded RNA viruses (dsRNA) of the *Totiviridae* family, have been identified. Among these, K28 represents the unique example of a yeast viral killer toxin that enters a sensitive cell by receptor-mediated endocytosis to reach its intracellular target(s). This review summarizes and discusses the most recent advances and current knowledge on yeast killer toxin K28, with special emphasis on its endocytosis and intracellular trafficking, pointing towards future directions and open questions in this still timely and fascinating field of killer yeast research.

## 1. Introduction

A killer phenotype in yeast was originally discovered in certain strains of the wine and brewery yeast, *Saccharomyces cerevisiae* (*S. cerevisiae*), and was first described in 1963 [1]. Characteristic of all “killer” yeasts is the production and secretion of a certain type of protein toxin (killer toxin) that is lethal to sensitive strains of the same or related yeast genera. Soon after the initial studies on the killer phenomenon in *S. cerevisiae*, it became evident that an infection with cytoplasmic inherited double-stranded (ds)RNA viruses is responsible for killer phenotype expression [2]. Interestingly, the occurrence of dsRNA viruses is not restricted to strains of *S. cerevisiae*, but rather, is widely distributed among various yeast species, including *Zygosaccharomyces bailii*, *Hanseniaspora uvarum* and *Ustilago maydis* [3,4]. Besides non-infectious yeast dsRNA viruses (also designated as virus-like particles, VLPs), the killer phenotype can also be chromosomally encoded (*Williopsis californica*) [5] or associated with linear dsDNA plasmids (*Klyveromyces lactis* and *Pichia acaciae*) [6]. The existence of dsRNA viruses in *S. cerevisiae* seems to be linked to the absence of RNA interference (RNAi), explaining why killer systems have so far only been found in RNAi-deficient yeast species, while RNAi-proficient yeasts did not develop killer strains during evolution [7]. While relatively little is known about the ecological relevance of killer toxin-producing yeasts, it has been proposed that yeast strains carrying killer viruses presumably possess a competitive advantage in the natural yeast habitat, in the battle for resources, by eliminating sensitive yeasts [8].

Based on the killing properties and the lack of cross-immunity, four different dsRNA-encoded killer types, namely K1, K2, K28, and Klus, have so far been identified in *S. cerevisiae* [9,10,11,12]. Each killer type shows killing activity against non-killer strains as well as killer strains of different killer types, while it is protected and immune against its own toxin. In nature, infected yeast cells only harbor a single copy of an M-dsRNA genome, whereby the coexistence of multiple M genomes with different killer specificities is excluded at the replicative level. Artificially, this limitation can be overcome by introducing cDNAs encoding killer toxins, K2 and K28, into a K1 strain, thereby artificially generating a triple killer strain that simultaneously expresses all three killer toxins and shows multiple toxin immunity [13]. To stably maintain a virally-encoded killer phenotype in yeast, two dsRNA genomes must be present in the cytoplasm of the infected host: an unsegmented 4.6 kb large L-dsRNA genome of the helper virus ScV-L-A and one of four smaller toxin-encoding M-dsRNA satellite viruses (ScV-M1, ScV-M2, ScV-M28, or ScV-Mlus) [10,14]. This review will mainly focus on ScV-M28 and its encoded killer toxin, K28.

## 2. K28 Phenotype: Origin, Genomic Organization and Viral Replication

**Figure 1:**
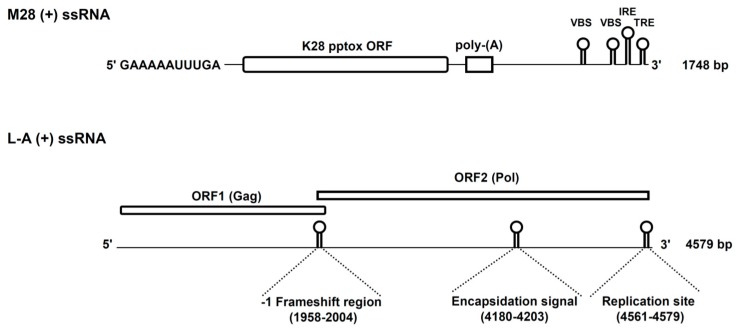
Genomic organisation of the L-A (+) and M28 (+) strands. The initial GAAAAA sequence at the 5′-end of the M28 (+) ssRNA represents a terminal recognition element (TRE) which is necessary for the initiation of transcription. M28 (+) ssRNA further contains the toxin-encoding K28 open reading frame (ORF), a poly(A)-rich region and potential 3′ elements which are required for in vivo RNA replication (IRE, internal replication enhancer; TRE, 3′ terminal recognition element) and packaging (VBS, viral binding side). Besides the two ORFs on L-A (+) ssRNA, the position of the −1 frameshift region, the encapsidation signal and the replication side for (−) strand synthesis are indicated in brackets. Reproduced and modified from [10,20], 2011 and 2013, American Society for Cell biology.

The first detailed analysis of the fundamental properties of the K28 killer phenotype in yeast was published in 1990 [11]. The phenotype was initially found in the *S. cerevisiae* wine strain 28, which gave the killer toxin its designation. As already shown for other killer toxins of *S. cerevisiae*, K28-producing killer strains harbor two different cytoplasmic persisting dsRNA genomes, which are separately encapsidated into virus-like particles (VLPs) [15]. The smaller 1.8 kb M-dsRNA genome of the ScV-M28 satellite/killer virus encodes the unprocessed K28 toxin precursor. The coding (+) strand of ScV-M28 contains a single open reading frame (ORF) that contains the genetic information for the unprocessed K28 precursor (also called preprotoxin, pptox) which likewise confers toxin immunity [14]. As classical satellite, ScV-M28 depends on a second virus (ScV-L-A) which functions as a helper virus required for stable maintenance and replication [16]. The linear L-A genome (4.6 kb) possesses two ORFs on its positive strand. The major 76 kDa capsid protein, Gag, is encoded by ORF1 and is required for proper dsRNA encapsidation and VLP formation. ORF2 encodes Pol, an RNA-dependent RNA-polymerase (RDRP) which is translated as a 180 kDa Gag-Pol fusion protein by a -1 ribosomal frameshift event [17,18,19]. Figure 1 summarizes the genomic organization and the coding capacity of the positive strands of ScV-M28 and ScV-L-A. 

For stable L-A helper and ScV-M28 satellite virus propagation within a killer cell, a subset of at least 28 nuclear *MAK* genes (*MAK1*, *MAK3-MAK27*, *PET18* as well as *SPE2*) is required in *S. cerevisiae* [3,21,22]. Mutations in any of these genes results in a rapid loss of M-dsRNA killer viruses, while an additional loss of L-A viruses is only observable in Δ*mak3*, Δ*mak10* and Δ*pet18* mutants [4]. Although the exact function and interplay of these genes in virus replication and maintenance is not fully understood, the concentration of free 60S ribosomal subunits seems crucial for efficient yeast virus propagation [23]. In contrast, recessive mutations in at least six chromosomal “super-killer” genes (*SKI*) lead to increased M-dsRNA copy numbers, associated with significantly enhanced killer toxin biogenesis and secretion [24,25]. For example, Δ*ski2* mutants secrete ten times more K28 toxin than wild-type killers [15]. Experimental results have indicated that *SKI* genes presumably possess a negative regulatory function for M-dsRNA virus replication and killer toxin expression [26,27,28]. 

**Figure 2:**
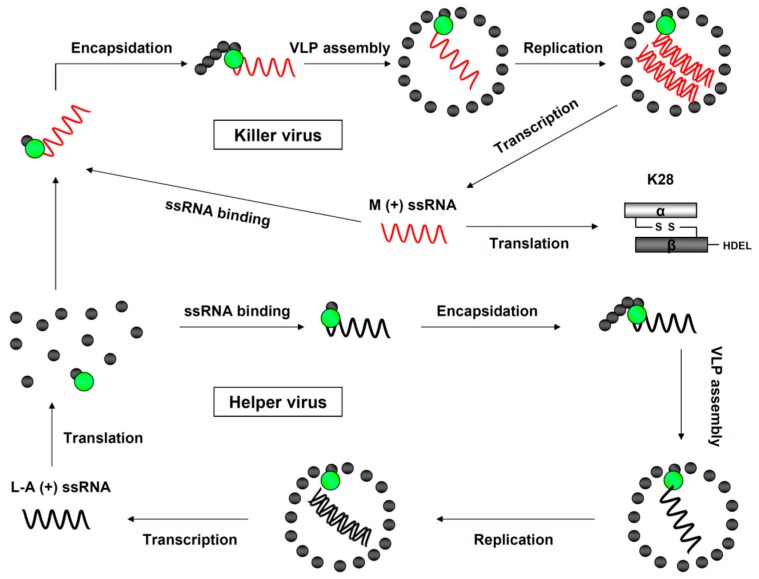
Replication cycle of L-A helper and M28 killer viruses in *S. cerevisiae*. The L-A replication cycle starts in vivo, with the transcription of L-A dsRNA into L-A (+) strands, via the transcriptase activity of the Gag-Pol fusion protein. After extrusion into the cytoplasm, the majority of L-A (+) strands are translated into the capsid protein, Gag (grey dot), while only 1–2% are converted into a Gag-Pol fusion protein (green-grey dot) by a −1 ribosomal frameshift event. Viral L-A (+) strands further interact with Gag-Pol, which triggers L-A particle assembly and encapsidation [39]. The protein composition of newly assembled ScV-L-A particles, each of them containing just a single L-A (+) strand, is identical to that of mature virions. In the final step, an L-A (−) strand is synthesized by the replicase activity of Pol, leading to a complete dsRNA genome within each virion. Replication of ScV-M is analogous to that of ScV-L-A. After in vivo M (+) strand synthesis and subsequent extrusion into the cytoplasm, M (+) strands are either translated into the unprocessed K28 toxin precursor or bound by Gag-Pol for subsequent encapsidation into virus particles. ScV-M replication finally finishes with the synthesis of an M (−) strand. Compared to L-A virions, two M-dsRNA copies can be present in a single M virion at the same time, due to their smaller genome size.

During VLP formation, a 39 nm icosahedral capsid, consisting of 60 Gag dimers and one or two Gag-Pol molecules, is assembled in the yeast cytoplasm [29,30,31,32]. So far, no extracellular route of infection has been described for dsRNA viruses in *S. cerevisiae*, as the non-infectious virions typically spread horizontally by heterokaryon formation or cell-to-cell mating [14]. As illustrated in Figure 2, the replication cycle of an L-A helper virus starts inside each virion with the generation of a single-stranded L-A plus strand ((+) ssRNA). After export into the yeast cell cytoplasm, (+) ssRNA transcripts are either translated into the viral proteins, Gag and Gag-Pol, or encapsidated into new virions [33]. Once the virus assembly is complete, the mature dsRNA genome is generated via Pol-mediated synthesis of the corresponding L-A minus strand [22,27,34,35,36]. The formation of toxin-coding M viruses occurs via the same steps as the L-A replication cycle, with the exception that two copies of the smaller M-dsRNA genome can be present in a single M virion. This phenomenon has been designated as “headful packaging”, which is likewise found in some DNA bacteriophages [37,38].

## 3. Viral Preprotoxin Processing and Toxin Secretion

**Figure 3:**
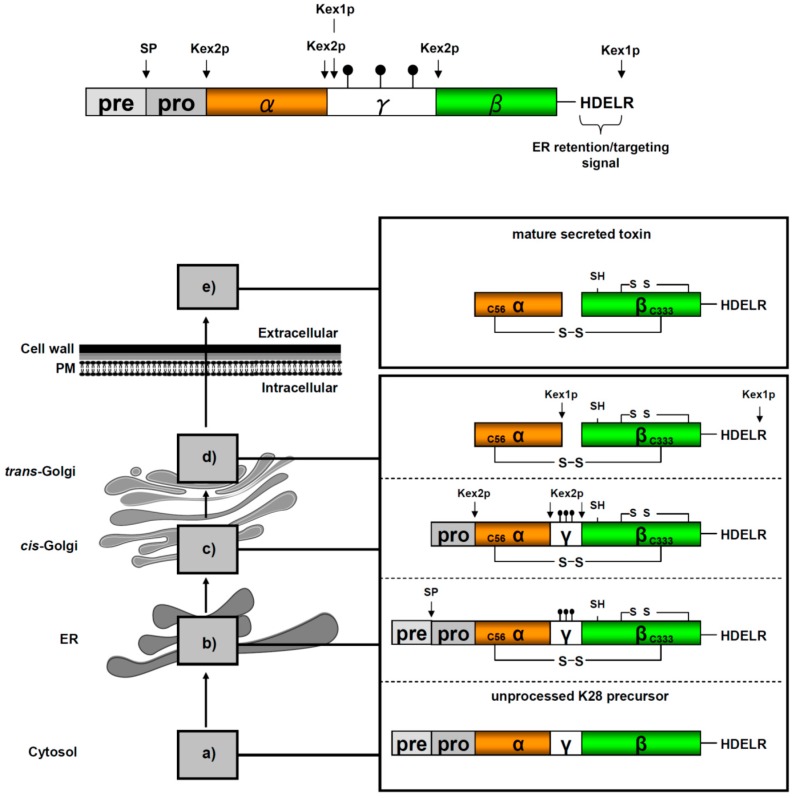
**Schematic K28 preprotoxin (pptox) structure and processing in the secretory pathway**. (**a**) pptox is completely translated by cytosolic ribosomes and thereafter post-translationally imported into the ER lumen via complex protein import machinery, including Sec61p, Sec71p, Sec72p Sec62p, Ssa1p and Ssa2p [40]; (**b**) Within the ER, the N-terminal signal peptide (pre-sequence) is removed by signal peptidase (SP) cleavage, the γ-subunit is *N*-glycosylated ( 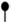 ) and the connecting disulfide (s-s) between α and β is formed by protein disulfide isomerase (Pdi1p); (**c**) In the *cis*-Golgi, the furin-like endopeptidase, Kex2p, removes the pro-sequence and γ-subunit which leads to a disulfide-bonded α/β heterodimer. Although the precise function of the pro-region is still not fully understood, it has been proposed to be important for proper post-translational pptox import into the ER lumen [40]. While the inter-chain disulfide in the heterodimer has clearly been demonstrated to be positioned between the single cysteine in α (Cys56) and Cys333 in β, the exact position of the cysteine residues in β that form the intra-chain disulfide and the single free thiol is still hypothetical [41,42]; (**d**) In the *trans*-Golgi, the C-terminus of the α-subunit as well as the C-terminal arginine of the β-subunit are removed by carboxypeptidase Kex1p cleavage, which unmasks the ER retention/targeting motif, HDEL, thereby converting the precursor in its biologically active conformation; (**e**) K28 is finally secreted as a 21 kDa heterodimer, whose β-C terminus carries a potential ER retention/targeting signal required for toxin uptake in a sensitive target cell [14].

In M28 virus-infected yeast cells, the cytoplasmic M (+) strand is translated into the unprocessed K28 toxin precursor (pptox) which is post-translationally imported, via Sec61p, into the yeast secretory pathway, where it undergoes extensive processing steps during toxin maturation and secretion [14,40]. A summary of the multiple enzymatic pptox processing steps in the ER and Golgi is shown in Figure 3.

Interestingly, major aspects of the enzymatic K28 pptox processing mirror pro-hormone conversion in mammalian cells [16,43]. As a naturally secreted protein, the unprocessed K28 precursor contains an N-terminal signal peptide for efficient pptox import into the ER lumen, followed by a pro-region (also designated δ-subunit) and the two toxin subunits α (10.5 kDa) and β (11.0 kDa) of mature K28 [14,16,40,44]. While the α-subunit represents the cytotoxic and cell killing polypeptide, the β-subunit mediates efficient K28 cell surface binding and toxin internalization. During pptox maturation in the secretory pathway, both toxin subunits are initially separated by an *N*-glycosylated γ-sequence which is believed to promote proper precursor folding and disulfide bond formation [45]. The four C-terminal amino acids (HDEL) in the fully processed β-subunit represent a classical ER retention motif [44,46], which is initially masked by a terminal arginine residue to allow efficient toxin transport through the secretory pathway and to prevent H/KDEL receptor-mediated toxin retrieval from the Golgi and retrograde transport to the ER [14]. As summarized in Figure 3, the toxin precursor is finally processed into a biologically active α/β heterodimer, whose subunits are covalently linked by a single disulfide bond [16,44]. In contrast to the former assumption that the cysteine residue, Cys340, in β is part of the inter-chain disulfide that joins both subunits in the mature K28 toxin [44], it was recently shown that the inter-chain disulfide is formed between the single cysteine in α (Cys56) and Cys333 in β. Within the cytotoxic α/β heterodimer, Suzuki et al. have postulated that the β-subunit contains a single free cysteine and an additional intra-chain disulfide between two of the remaining cysteines in β (C292, C307 and C340) [42].

## 4. Cell Wall Binding and Endocytosis of K28

**Figure 4:**
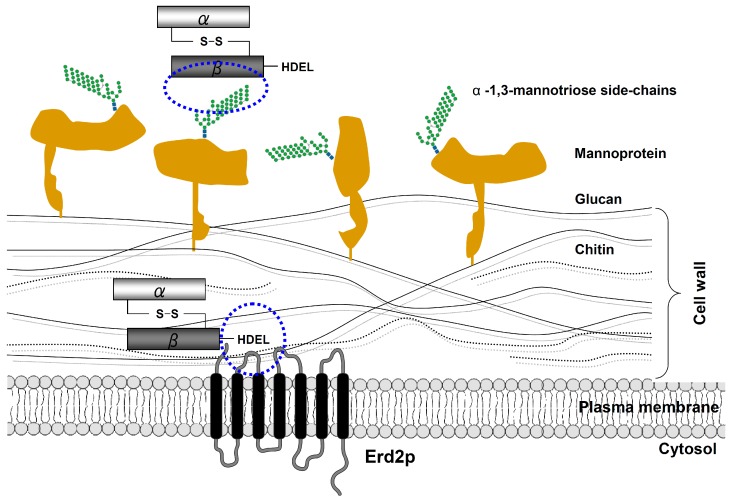
Schematic outline of K28 cell surface binding and internalization. Initially, the β-subunit of the mature K28 toxin rapidly binds in an energy-independent process to primary mannoprotein receptors in the yeast cell wall. In a second energy-dependent step, K28 penetrates the cell wall and subsequently interacts with the plasma membrane (PM)-localized pool of the yeast H/KDEL receptor Erd2p [14]. This interaction is mediated by the C-terminal HDEL motif of the toxin’s β-subunit, which finally triggers receptor/toxin complex internalization by clathrin-mediated endocytosis.

Bacterial A/B toxins, such as cholera toxin or Shiga toxin, initially bind to specific receptors at the mammalian cell surface to subsequently enter cells by endocytosis and reach their final target in the cytosol [47]. In the case of K28, early studies in the nineties have demonstrated that a specific primary receptor in the yeast cell wall allows rapid K28 absorption via an energy-independent binding mechanism (Figure 4). Later on, α-1,3- and/or α-1,2-mannotriose side-chains of a 185 kDa cell wall mannoprotein were identified as primary K28 receptor and cell wall binding sites [48,49,50,51]. In line with this, mutations in chromosomal yeast genes encoding mannosyltransferases, including Mnn1p and Mnn5p, which catalyze mannoprotein side-chain synthesis and structure, prevent toxin binding and, consequently, result in K28-resistance [49]. However, besides alterations in cell wall mannoproteins, a large number of additional deletion mutants affecting protein mannosylation, lipid biogenesis and GPI anchor biosynthesis have likewise been shown to negatively affect K28 cell surface binding. Accordingly, the primary process of toxin binding to sensitive target cells seems much more complex than originally thought, depending on a variety of factors which ensure optimal cell surface binding of the K28 toxin [52]. 

In contrast to the ionophoric killer toxins, K1 and K2 [53,54,55], K28 is taken up via receptor-mediated endocytosis. Consequently, yeast deletion mutants, such as Δ*end3* and Δ*end4*, with severe defects in early steps of both fluid-phase and receptor-mediated endocytosis, become toxin resistant, due to a block in K28 uptake from the cell surface [56]. Since yeast cell spheroplasts, lacking an intact cell wall and primary toxin receptors, are still phenotypically K28 sensitive, the existence of a secondary toxin receptor at the level of the yeast plasma membrane (PM) was proposed for a long time, but only recently identified. In this respect, it was demonstrated that a minor fraction of PM-localized Erd2p, the yeast H/KDEL receptor, is responsible for K28 plasma membrane binding and internalization (Figure 4). Thereby, Erd2p interacts with the C-terminal HDEL motif of the β-subunit, thereby triggering toxin uptake by receptor-mediated endocytosis. The current model of host cell intoxication by K28 is supported by two major observations: (i) a K28 variant, lacking a C-terminal HDEL motif in β is not taken up by sensitive yeast cells and (ii) yeast cells lacking Erd2p are unable to internalize cell bound killer toxins [57]. Although it is not yet clear how the resulting Erd2p/K28 receptor/toxin complex is recognized by the endocytosis machinery and how its internalization is regulated, it is important to note that efficient K28 uptake not only requires a correct protein/lipid environment in the plasma membrane, but also a functional AP2 complex. Mutations in individual subunits of the yeast AP2 complex completely block K28 internalization and thus, prevent toxin-mediated cell killing. In this context, the AP2 complex most likely acts as a specific adaptor to connect the receptor/toxin complex with the clathrin coat of the endocytosis machinery [52]. Interestingly, receptor (mono)ubiquitylation, via Ubc4p (E2) and Rsp5p (E3), seems likewise required for efficient K28 endocytosis, since a chromosomal deletion of *UBC4* and/or temperature-sensitive mutations in *RSP5*, significantly reduce the amount of internalized toxin [58]. However, further experiments are needed to identify the underlying mechanisms that regulate K28 endocytosis.

## 5. Intracellular K28 Trafficking from the Plasma Membrane to the ER

**Figure 5:**
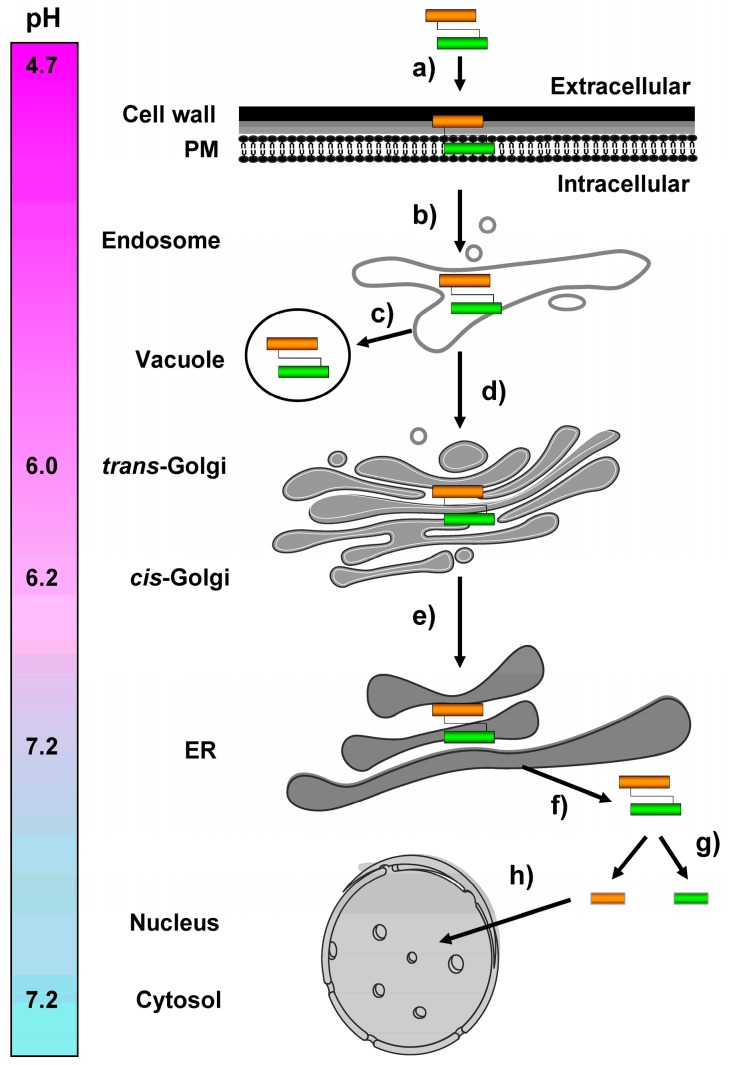
Schematic overview of retrograde toxin trafficking routes in a K28 sensitive target cell. (**a**) Biologically active K28 toxin (α-subunit shown in orange, β-subunit in green) initially binds to primary receptors in the yeast cell wall and then interacts with a secondary receptor, the yeast H/KDEL receptor, Erd2p, at the level of the plasma membrane; (**b**) After clathrin-mediated endocytosis, a minor fraction of internalized toxin/receptor complexes is targeted to the vacuole and degraded (**c**), while toxin/receptor complexes that escaped degradation are transported in a retrograde manner to the pH-neutral ER lumen, where the receptor/cargo complexes dissociate and release K28 (**d** + **e**). The natural pH gradient between the extracellular killer yeast environment (pH 4.7) and the intracellular compartment of the ER (pH 7.2) presumably prevents premature toxin dissociation from its receptor (Erd2p) and likewise prevents spontaneous formation of inactive K28 oligomers (see also Chapter 7 below); (**f**) In contrast to, for example, cholera toxin, ER exit of K28 occurs in its oxidized and α/β heterodimeric conformation and is likely gated by the major protein import/export channel in the ER membrane, Sec61p [56,58]; (**g**) Within the cytosol, the heterodimeric toxin dissociates into its subunits: while the β-subunit is ubiquitylated and proteasomally degraded; (**h**) the α-subunit enters the nucleus and finally kills, by causing an irreversible G1/S cell cycle arrest and inhibiting DNA synthesis.

In the current model, K28/Erd2p complexes are internalized and targeted to an early endosomal compartment, from where the receptor/cargo complex travels the secretion pathway in reverse and finally exits the ER lumen to enter the yeast cytoplasm. Due to pH-dependent binding of the toxin’s ER retention/targeting motif (HDEL) and Erd2p [59], it is conceivable that K28 initially binds to PM-localized Erd2p receptors in the mildly acidic milieu (pH ~4.7) of the extracellular environment. The complex remains associated during endocytosis and retrograde transport, through the likewise mildly acidic environment of the endosomal and Golgi compartments (pH ~6.0–6.2) until it reaches the pH neutral (pH ~7.2) ER lumen (Figure 5). So far, it is not precisely known how the endosome-to-Golgi transport of the receptor/toxin complex is regulated and how its vacuolar transport and degradation is largely prevented. The hypersensitive K28 phenotype of yeast mutants defective in vacuolar HOPS tethering complex components, however, indicates that a certain fraction of the toxin/receptor complex is indeed targeted to the vacuole and degraded [52]. It therefore can be assumed that defects in the vacuolar degradation pathway result in increased toxin traffic to the ER, a phenomenon which has also been described for bacterial and plant A/B toxins, such as Shiga-like toxins and ricin [60]. 

Over the years, several studies identified a number of yeast genes whose gene products are required for proper anterograde and retrograde transport of cellular proteins and likewise are important for K28 trafficking [52,56,58]. Unfortunately, phenotypic analysis of yeast mutants defective in the corresponding transport genes is not trivial, since different possibilities exist to explain how these mutations could affect toxin transport. Defects in general protein transport can affect protein traffic, within and through the secretory pathway, eventually leading to an altered amount of protein transport to the plasma membrane, which could lead to a decrease in the Erd2p copy number at the cell surface and/or indirectly affect retrograde K28 transport. On the other hand, direct effects on retrograde toxin transport could also lower the total number of toxin molecules that reach the ER and/or cytosol. It will be a challenging future task to clarify in which ways such mutations affecting K28 transport through the endomembrane system.

## 6. ER-To-Cytosol Retrotranslocation

**Figure 6:**
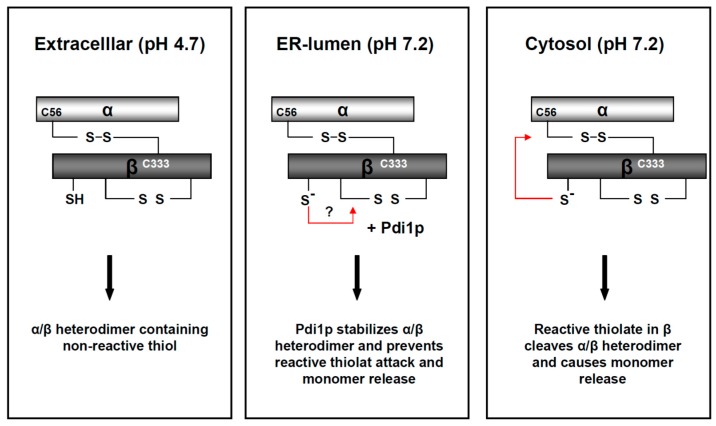
Model of pH-driven and Pdi1p-dependent thiol rearrangements in the K28 heterodimer during its retrograde transport to the cytosol. In the natural habitat and extracellular environment of a K28 killer yeast, the mildly acidic pH of 4.7 stabilizes the biologically active K28 toxin and keeps it in a heterodimeric conformation. During host cell intoxication, the α/β heterodimer faces a continuous increase in intra-compartmental pH. The neutral pH leads to fast deprotonation of free sulfhydryls in the β-subunit and subsequently causes the formation of inactive K28 trimers, tetramers and oligomers. Due to the presence of the chaperone, Pdi1p, in the ER-lumen, these disulfide bond rearrangements are efficiently prevented, in vivo and in vitro, ensuring ER exit of the heterodimeric toxin. At the neutral pH of the yeast cell cytosol, the intra-chain disulfide is cleaved through nucleophilic attack of a reactive thiol in β, which finally releases the monomeric α-subunit. This model is reproduced and modified from [42], 2017, American Society for Cell biology.

Once the K28 toxin reaches the ER-lumen, it has to undergo conformational changes to allow its export into the cytosol. Interestingly, and in contrast to many other A/B toxins, the subunit connecting disulfide in the α/β toxin remains intact during retrotranslocation and gets reduced in the cytosol. Heiligenstein et al. further demonstrated that certain chaperones in the ER lumen, including Kar2p, protein-disulfide isomerase (Pdi1p), the Hsp40 chaperones Jem1p and Scj1p, as well as a proper maintenance of Ca^2+^ homeostasis in the ER, are important factors for K28 export into the cytosol. In contrast, neither ubiquitylation nor ER-associated degradation (ERAD) machinery are required for efficient K28 retrotranslocation [58]. This mechanism contrasts K28 from other A/B toxins (e.g., cholera toxin or ricin) that have been shown to parasitize components of the ERAD machinery to exit the ER after disulfide bond reduction [58,61,62,63,64]. For these toxins, it has been assumed that the release of the corresponding cytotoxic subunit, before and/or during ER-to-cytosol transport, functions as an initiation signal for toxin retrotranslocation from the ER. Most recently, a model of K28 host cell intoxication was postulated, in which the intracellular pH gradient from the PM to the ER lumen (ranging from mildly acidic to neutral pH conditions, Figure 5) activates and rearranges the thiols in the α/β heterodimer [42]. In the case of K28, this pH-triggered rearrangement leads to the formation of inactive toxin trimers, tetramers and oligomers in vitro. Interestingly, this oligomer formation seems prevented in vivo by the activity of the ER chaperon, Pdi1p, keeping K28 in its heterodimeric conformation [42]. It has been speculated that Pdi1p induces some additional, and so far unknown, structural changes in K28, which ensure toxin retrotranslocation into the cytosol on the oxidized K28 heterodimer [58]. In the absence of Pdi1p, the activated and rearranged thiols would generate the driving force for premature release of the cytotoxic α-subunit from the heterodimer [42]. This novel mechanism represents a unique A/B toxin strategy to penetrate and finally kill a target cell. Nevertheless, further efforts are needed to identify the specific structural changes in K28 which ensure efficient toxin export into the cytosol; Figure 6 illustrates a simplified current model of K28 structural rearrangements, during intracellular transport.

## 7. Mode of K28 Toxicity

In the natural yeast habitat of limiting nutrients, K28 killer strains secrete mature K28 toxin into the extracellular environment to outcompete sensitive yeasts. After receptor-mediated toxin uptake via Erd2p and retrograde transport through the secretory pathway into the cytosol of a sensitive yeast cell, the cytotoxic α-subunit is released from the heterodimer and enters the nucleus by passive diffusion, due to its low molecular weight of 10.5 kDa. In this respect, in vivo toxicity of the α-subunit can be significantly enhanced by the addition of a C-terminal nuclear localization sequence (NLS) [14], while it is artificially reduced in an α-variant containing a nuclear export sequence (NES) (unpublished data). However, the underlying mechanisms of how K28-α efficiently enters the nucleus and finally kills are still largely unknown. Nuclear proteins, which have already been identified as interaction partners of K28, belong to cellular proteins that are centrally involved in cell cycle control, gene expression and chromatin remodeling. In addition, it has been shown that mutations in yeast genes encoding subunits of the mediator complex, RNA polymerase II, and/or components of the SIR-, SWR1 and ASTRA complexes, all dramatically reduce K28 sensitivity in their corresponding genetic background, however, their specific roles and interplays are still unknown [52].

With respect to the mode of K28-induced cell killing, two different dose-dependent phenotypes have been identified: low killer toxin concentrations (<1 pM) induce an apoptotic cell death response in toxin-treated cells, with typical markers, such as DNA fragmentation, chromatin condensation, and phosphatidylserine exposure, at the outer plasma membrane [65]. Furthermore, apoptotic cell killing by K28 was shown to depend on the presence of yeast caspase (Yca1p) and the generation of reactive oxygen species (ROS) [66]. This scenario of cell killing closely reflects the natural situation of low K28 concentrations in a yeast habitat and, thus, toxin-induced apoptosis seems a crucial, if not major mechanism by which K28-secreting killer strains outcompete and kill sensitive yeasts. In contrast, under laboratory conditions of high toxin concentrations (≥10 pM), cell killing occurs through necrosis, during which cells arrest at the G1/S boundary of the cell cycle with a medium-size bud and a single nucleus in the mother cell with pre-replicated (1n) DNA [67]. Furthermore, inhibition of DNA synthesis was also observed under high toxin concentrations [48,65]. Interestingly, another yeast killer toxin (PMKT2) from *Pichia membranifaciens* shows nearly the same killing phenotype after application of high and low toxin doses, while both the intoxication mechanism(s) and cellular target(s) of PMKT2 are completely different from K28 [68]. Hence, apoptosis is not only triggered during the pathogenesis of various virus infections in mammals, it is likewise activated by different yeast killer toxins, to eliminate target cells [69]. In sum, the killer toxin K28 has developed an elegant, simple and unique strategy to intoxicate and kill sensitive cells by targeting evolutionary highly-conserved proteins with essential cell functions. Moreover, this strategy largely avoids the occurrence of spontaneous mutations resulting in toxin resistance, as most gene products that are parasitized by K28 are genetically encoded by essential yeast genes.

## 8. Toxin Immunity

**Figure 7:**
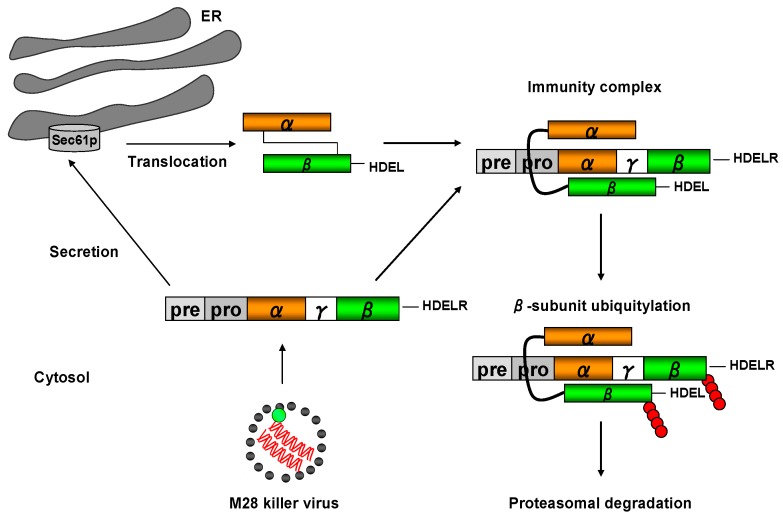
Model of the protecting toxin immunity mechanism in a K28 killer yeast. After toxin internalization and retrotranslocation of mature K28 from the ER into the cytosol, a cytosolic immunity complex is formed between the re-internalized α/β toxin and the unprocessed toxin precursor (pptox) which is encoded by the M28 killer virus and post-translationally imported into the ER lumen. Within each K28/pptox complex, the β-subunit of pptox and mature α/β toxin is poly-ubiquitylated (●) and degraded by the proteasome, thereby protecting a K28 killer cell against the lethal action of K28. In addition, free cytosolic and non-ubiquitylated pptox can enter the secretory pathway for enzymatic processing and toxin maturation, resulting in the secretion of biologically active K28.

As toxin cell binding, uptake and retrograde transport in a K28-secreting killer cell occurs in exactly the same way as in a sensitive target cell, K28 killer cells must be protected against their own toxins. This represents a major difference to A/B toxin-producing bacteria, such as *Vibrio cholerae* (cholera toxin) or *Shigella dysenteriae* (Shiga toxin), which secrete protein toxins that selectively kill eukaryotic cells, making a protecting immunity mechanism dispensable for a prokaryotic toxin-producing microorganism [47]. In K28 killer strains, functional immunity is manifested by an intrinsic mechanism, which allows rapid degradation of the internalized α/β toxin after retrotranslocation into the host cytosol (Figure 7).

Once K28 reaches the cytosol, it forms a complex with the fully translated pptox precursor that has not yet been post-translationally imported into the ER [14]. Thus, K28 immunity is severely impaired when the natural N-terminal pre-sequence of K28 is replaced by the co-translationally active signal peptide from K1 pptox [40]. Within the immunity complex, the β-subunit(s) of mature K28 and K28 pptox are selectively poly-ubiquitylated and immediately targeted for proteasomal degradation. Changes in cellular ubiquitin homeostasis, e.g., induced by overexpressing mutant ubiquitin or by blocking deubiquitylation, severely impair functional toxin immunity and result in a suicidal K28 phenotype [40]. Due to an excess of non-complexed K28 precursor molecules in the killer strain cytosol, a pptox fraction is likewise imported into the ER lumen, subsequently processed and finally secreted as an α/β heterodimeric and biologically active toxin. This elegant strategy guarantees that K28 killer strains are efficiently protected against their own toxins and, consequently, provides an evolutionary growth advantage over sensitive yeast strains. Since the molecular mechanism of toxin immunity has so far only been identified for K28, it is still a mystery how toxin immunity is realized in other killer toxin secreting yeasts.

## 9. Open Questions and Future Perspectives

In the last decades, intensive research in the field of virus-infected killer yeasts has discovered the fundamental principles underlying killer phenotype expression, maintenance and transmission to progeny cells. The knowledge of K28 precursor processing has further helped to understand the basic mechanisms of A/B toxin and pro-hormone conversion in higher eukaryotes [14]. Moreover, recent data has generated a more detailed picture of toxin internalization and intracellular trafficking from the plasma membrane to the host cell cytosol [52,57]. The finding that pH-triggered disulfide rearrangements in the yeast killer toxin, K28, are crucial for host cell killing, represents a further piece in the puzzle to uncover the complete intoxication mechanism of this unique killer toxin [42]. However, some parts of the K28 intoxication process, including the precise mode of cell killing, are still poorly understood or simply unknown. Although the dose-dependent effects of K28-treated cells are well described (G1/S cell cycle arrest at high K28 concentration, apoptosis at low toxin concentration), the underlying mechanisms and cellular targets required for K28-mediated cell killing are still largely unknown. In future studies, it therefore will be a great benefit to successfully implement new methods and techniques, such as biochemical cross-linking and APEX proximity labelling, to identify the cellular target(s) of K28. 

Another important issue in future yeast killer toxin research will be to resolve the three-dimensional structure of the toxins. Up to now, regrettably, no such information has been available for any of the *S. cerevisiae* killer toxins, in the literature. Furthermore, an in silico analysis did not show any homology to other known proteins in current databases. Another critical issue that K28 research needs to address is the isolation and purification of high yields of biological active toxin which would be needed for crystallization and X-ray analysis or NMR spectroscopy. Commonly used affinity purification methods are usually performed at higher pH (pH > 6), which irreversibly inactivates K28 during the purification process (unpublished data). Using the methylotrophic yeast, *Pichia pastoris*, which was demonstrated to be a suitable host for the successful expression and secretion of the cytotoxic K28 toxin [70], might be a fruitful strategy for high-level K28 production.

Our current knowledge of killer toxin cell binding and intracellular trafficking mainly arises from genetic screens for yeast mutants with altered K28 sensitivity. To overcome this methodical limitation, the construction of fluorescent and biologically active K28 variants is essential to monitor in vivo intoxication in real time, through live cell imaging and high-resolution microscopy. Until now, these attempts have mainly failed, due to the toxin’s sensitivity to pH changes and/or epitope extensions of the cytotoxic α-subunit, which has always led to the production of non-toxic K28 variants (unpublished data).

In addition to the aforementioned aspects, the following new questions have arisen from more recent studies on the yeast K28 toxin: What are the exact structural changes mediated by Pdi1p during ER-to-cytosol retrotranslocation of the heterodimeric toxin? How does K28α enter the nucleus and which components are required for retrograde toxin trafficking from endosomes through the Golgi to the ER. Besides its general importance in deepening the overall understanding of basic processes in eukaryotic cell biology, the K28 toxin has the potential to be used in various fields of applied biotechnology and biomedicine. In this context, for instance, triple K1/K2/K28 killer yeast strains have been constructed that might be useful as biocontrol agents and starter cultures in wine and beer fermentation, to prevent or eliminate contamination with undesired yeasts, like *Candida* or *Pichia* [4,13,71]. Furthermore, and directly related to the most recent findings on K28 in intoxication and intracellular transport, it might be conceivable for future studies to use the β-subunit of K28 as specific transport vehicle for the targeted import of recombinant proteins or polypeptide-based therapeutics into a eukaryotic target cell, thereby ensuring more efficient transport of chimeric K28β/protein fusions from the plasma membrane to the cytosol and to largely prevent or minimize vacuolar/lysosomal degradation. 

## 10. Conclusions

Many new aspects of fundamental cellular processes have been learned over the past decades by studying virus-infected killer yeasts and analyzing intracellular toxin transport, maturation and secretion. In the case of K28, a wide range of important cellular processes, including receptor-mediated endocytosis, retrograde protein transport, ER-to-cytosol retrotranslocation, proteasomal degradation, and even apoptotic host-cell responses have been investigated in more detail. Future studies will greatly benefit from the continuous and fast development of high resolution imaging techniques and novel biochemical tools that can help to fully uncover the K28 intoxication mechanism and answer open questions in this still timely and fascinating research field.
