# Correction: Becker, B. et al. Yeast Killer Toxin K28: Biology and Unique Strategy of Host Cell Intoxication and Killing

**DOI:** 10.3390/toxins10040132

**Published:** 2018-03-23

**Authors:** Björn Becker, Manfred J. Schmitt

**Affiliations:** Molecular and Cell Biology, Department of Biosciences and Center of Human and Molecular Biology (ZHMB), Saarland University, D-66123 Saarbrücken, Germany; bjoern_becker2@gmx.de or b.becker@microbiol.uni-sb.de

The authors are sorry to report that Figure 3 in their published paper [1] was incorrect. The correct figure should be: 
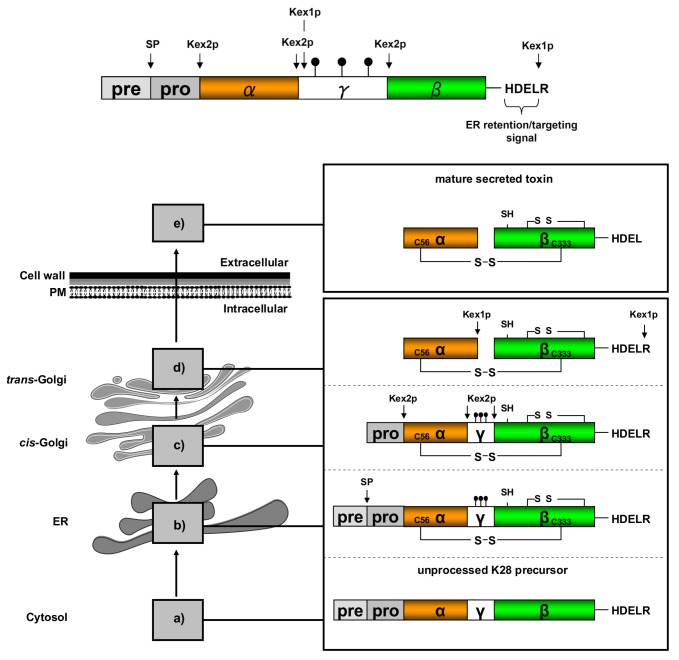


We apologize for any inconvenience caused to the readers.
